# Dapsone Hypersensitivity Syndrome in Leprosy

**DOI:** 10.4269/ajtmh.25-0484

**Published:** 2025-10-21

**Authors:** Harish Kumar Sagar

**Affiliations:** Clinical Division, ICMR-National JALMA Institute for Leprosy and other Mycobacterial Diseases, Agra, India

Leprosy is a chronic granulomatous disease that often poses a challenge due to its underlying stigma. The WHO recommends multidrug therapy (MDT) that consists of a three-drug regimen (including rifampicin, dapsone, and clofazimine) for all leprosy patients. This therapy should be administered for 6 months for paucibacillary leprosy and 12 months for multibacillary leprosy.^1^

Dapsone (diaminodiphenyl sulfone [DDS]) is a sulfone-derived drug that causes both dose-dependent hemolytic anemia and methemoglobinemia, as well as dose-independent dapsone hypersensitivity syndrome (DHS).^2^

**Figure 1.**
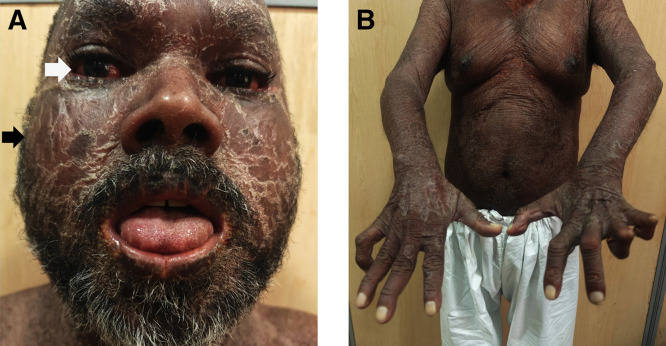
Dapsone hypersensitivity syndrome. (**A**) Black pointer: scaly lesions; white pointer: sub-conjunctival hemorrhages. (**B**) Ulnar claw hand deformity.

A 45-year-old man presented with well-defined to ill-defined, hypopigmented, anesthetic patches over his limbs and back that had persisted for 2 years. After a clinical evaluation was conducted at a primary health center, a diagnosis of borderline leprosy was made, and MDT was initiated. Four weeks after starting treatment, the patient developed a high-grade fever (39.4°C), extensive scaly lesions on his skin, icterus, pruritus, subconjunctival hemorrhages, angular cheilitis, and oral ulcers (Figure 1A).

**Figure 2.**
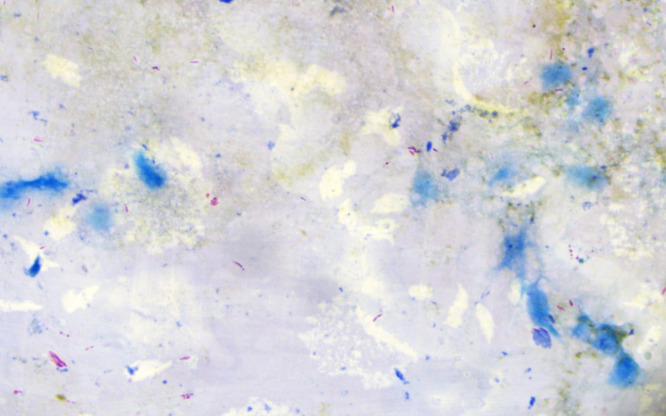
Slit skin smear (Ziehl–Neelsen stain; 100×). The solid staining bacilli represent live bacilli, whereas the granular bacilli represent dead bacilli.

A neurological examination revealed thickening of the bilateral ulnar, radial cutaneous, and lateral popliteal nerves. A bilateral ulnar claw hand deformity was observed (Figure 1B). A slit skin smear examination yielded a bacteriological index of ≥3 (Figure 2).

Laboratory results revealed mild anemia (hemoglobin: 9 g/dL), eosinophilia (absolute eosinophil count: 1,125 cells/*µ*L), and abnormal liver function tests (total bilirubin: 3.83 mg/dL; alanine transaminase: 543 U/L, aspartate aminotransferase: 253 U/L, alkaline phosphatase: 150 U/L). Glucose-6-phosphate dehydrogenase levels were within normal limits. A peripheral blood smear did not reveal any hemoparasites. Urinalysis results are also within normal limits.

**Figure 3.**
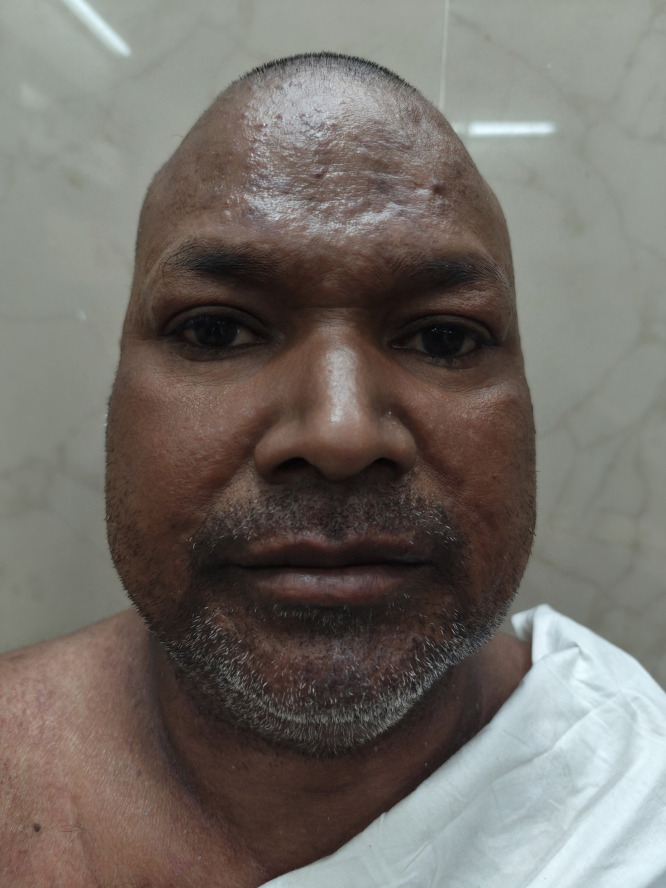
Dapsone hypersensitivity syndrome after 2 weeks of supportive treatment.

Considering the triad of high-grade fever, rash, and systemic involvement, a diagnosis of DHS was established. Intramuscular betamethasone (4 mg, twice daily) was initiated and gradually tapered to oral prednisolone (0.6 mg/kg/day). Multidrug therapy was discontinued because of DHS, along with the clinical presumption that rifampicin-induced hepatitis and clofazimine-induced ichthyosis were likely causes. After 2 weeks of supportive treatment, the symptoms and scaly lesions resolved (Figure 3). Subsequently, MDT was reintroduced with a modified regimen (day 1: rifampicin 600 mg, ofloxacin 400 mg, and clofazimine 300 mg, days 2–28: ofloxacin 400 mg and clofazimine 50 mg administered daily). This regimen excluded dapsone and incorporated a second-line drug (ofloxacin), which was well tolerated.

The classic description of DHS is a “triad” of fever, rash, and systemic involvement that typically develops ∼6 weeks after dapsone initiation. Cutaneous manifestations include erythroderma (exfoliative dermatitis), erythematous maculopapular eruptions, erythema multiforme, toxic epidermal necrolysis, and eruptions similar to those seen in Stevens–Johnson syndrome.^3^ If not recognized promptly, DHS may lead to irreversible organ damage and has an estimated mortality rate of 9.9–12.5%.^4^^–^^6^ Early detection and appropriate management are essential for a favorable prognosis.
